# Lessons learned from developing a statewide research data repository to combat the opioid epidemic in Alabama

**DOI:** 10.1093/jamiaopen/ooac065

**Published:** 2022-08-01

**Authors:** Matthew Hudnall, Dwight Lewis, Jason Parton, Christopher Sellers

**Affiliations:** Information Systems, Statistics, and Management Science, University of Alabama, Tuscaloosa, Alabama, USA; Management, University of Alabama, Tuscaloosa, Alabama, USA; Information Systems, Statistics, and Management Science, University of Alabama, Tuscaloosa, Alabama, USA; Alabama Department of Mental Health, Montgomery, Alabama, USA

**Keywords:** opioid, dashboard, data, integration, best practice

## Abstract

Many states are continuing to struggle with opioids and other commonly abused drugs. Alabama, being the highest opioid prescription per-capita state since 2012, has pulled together state agencies, private companies, academia, and community organizations to form a data council and repository to provide unified insights and information to the public and partner stakeholders. The lessons learned in constructing this data environment are documented so that other states and organizations can benefit from the challenges and success that Alabama has experienced. The centralized data repository consists of almost a dozen data streams from public agencies and private companies. The data are transformed and linked within the repository to provide geo-temporal linkages between data sources. The data are stored in a secure multi-tiered environment in a Microsoft SQL Server database, de-identified, aggregated, and then published to a public web portal for open consumption. The public-facing website from the project successfully integrates multiple disparate data sources into a common platform for streamlined and cohesive data communications. Drug addiction cannot be easily quantified, viewed, or otherwise examined when only looking at a portion of society. By bringing together multiple data sources and linking them, a more clear picture of trends, influences, and metrics can be obtained. A statewide drug use data partnership between public and private entities is both possible as well as beneficial to all parties involved. Items like legal contracts can inhibit data sharing, but certain best practices can help scale and streamline multiple agreements.

## INTRODUCTION

The challenges of combating the opioid epidemic in the United States are well-documented. Despite extensive efforts and investment of financial resources to mitigate this challenge, statistics suggest that adults are more likely to die in their lifetime from an opioid overdose than a motor vehicle crash.^1^ While evidence suggests that recent policy efforts have reduced the misuse and abuse of prescription opioids,^2^ deaths involving synthetic opioids like fentanyl have increased a staggering 645% since 2010.^3^ In the state of Alabama, the opioid crisis is a public health and economic emergency that is prematurely eroding the quality of life for far too many across multiple societal sectors.^4^ In 2012, Alabama was recognized as having the nation’s highest per-capita opioid prescription rate with 143.8 prescriptions per 100 residents.^5^ While this rate appears to be decreasing each year in Alabama, the state remains the highest per capita opioid prescribing state^5^ and is decreasing at a slower rate compared to most other states. Given these considerations, new approaches to addressing this epidemic are warranted, and leveraging time-sensitive data infrastructures hosted by various clinical and government stakeholders may be the nation’s best opportunity to get ahead of the opioid epidemic.^6^

## BACKGROUND AND SIGNIFICANCE

In 2017, Alabama’s governor established the Alabama Opioid Overdose and Addiction Council, which developed a comprehensive strategic plan to abate the state’s opioid crisis. Inefficient data dissemination throughout the affected stakeholder tiers (ie, public, law enforcement, prescribers/dispensers, treatment, law enforcement, and medical examiners) was identified by the council as a significant barrier to developing effective strategies to improve opioid rates within Alabama. The council focused on this barrier because data on drug abuse, treatment, and public safety outcomes are usually structured for the operations of each stakeholder and rarely designed to support needed analytics to facilitate the planning management of interdisciplinary policies. As such, the council determined a need for a centralized data repository (DrugUse-CDR), which combines data from multiple stakeholders to track both longitudinal as well as geospatial propagation of opioids in Alabama.^7^ In 2018, The University of Alabama’s Institute of Data and Analytics (IDA) was selected as the academic partner and implementer of DrugUse-CDR, as well as the manager of the public-facing web URL of druguse.alabama.gov.

There are both a data advisory board and a data governance council for the CDR. The advisory board is made up of a broad spectrum of constituents that has a heavy focus on data users/consumers, such as epidemiologists, grant writers, and analysts from partner agencies and community organizations that rely on timely data for interventions, grant proposals, and policy formulation. The council is made up of a representative from each agency that has a signed data agreement and shares data with the CDR. The advisory board provides suggestions and input, and the governance council codifies the suggestions and provides additional input. While each agency member of the council has an equal vote in regards to policy, a single member can veto any usage of the data for which they represent. This mechanism of keeping authority over the usage of data was a key step in assuring agency buy-in. As new agencies and partner organizations are added, new seats on the council are added to ensure that the new agencies maintain control over their data and have a say in the usage and formatting of the overall system.

## EARLY PHASE IMPLEMENTATION OF DRUGUSE-CDR

The core funding for DrugUse-CDR was awarded to the Alabama Department of Mental Health through a Department of Justice Comprehensive Opioid Abuse Site-based Program (COAP) grant. In its early stages, the overarching goal of DrugUse-CDR was to create an extensible data repository and secure process for stakeholders to acquire and make better use of data resources to address opioid use and misuse in Alabama. DrugUse-CDR requires an unprecedented level of cooperation and data sharing between partner state agencies, which poses unique challenges with data security and the protection of health information. Therefore, the IDA was initially tasked with developing and implementing the needed data infrastructure of this highly sensitive data for the first 2 years of the program.

The geodesign process provided the DrugUse-CDR project with a theoretical foundation for project implementation. The IDA elected to use Carl Steinitz’s geodesign process^8^ as the framework by which DrugUse-CDR’s infrastructure was built. Commonly used in regional planning, the geodesign process is a compilation of methods and activities that use data to “model” the natural environment, which is thereafter used for the creation of feasible solutions to address problems or objectives. What distinguishes this framework from other methods commonly used by academic researchers is that the geodesign process is centered on the involvement of all key stakeholders.^9^ This conceptual framework was preferred over others because some stakeholders expressed frustrations with past academic collaborations where their opinions were not taken seriously. Also, the geodesign process was preferred since DrugUse-CDR has a strong geospatial focus.

What makes this project more unique compared to other academic and government collaborations is that the academic researchers in this project are more focused on the science of data utilization, and the community stakeholders are focused on clinical outcomes. To that end, state government departments are steering intervention stages of the geodesign process, and the academic researchers are focused on the assessment stages of the geodesign process. Though both parties are involved in all aspects of the project, the stakeholders serve as the experts in addiction, while the academic researchers awarded the grant are adept in data management and intelligence.

**Figure 1.**
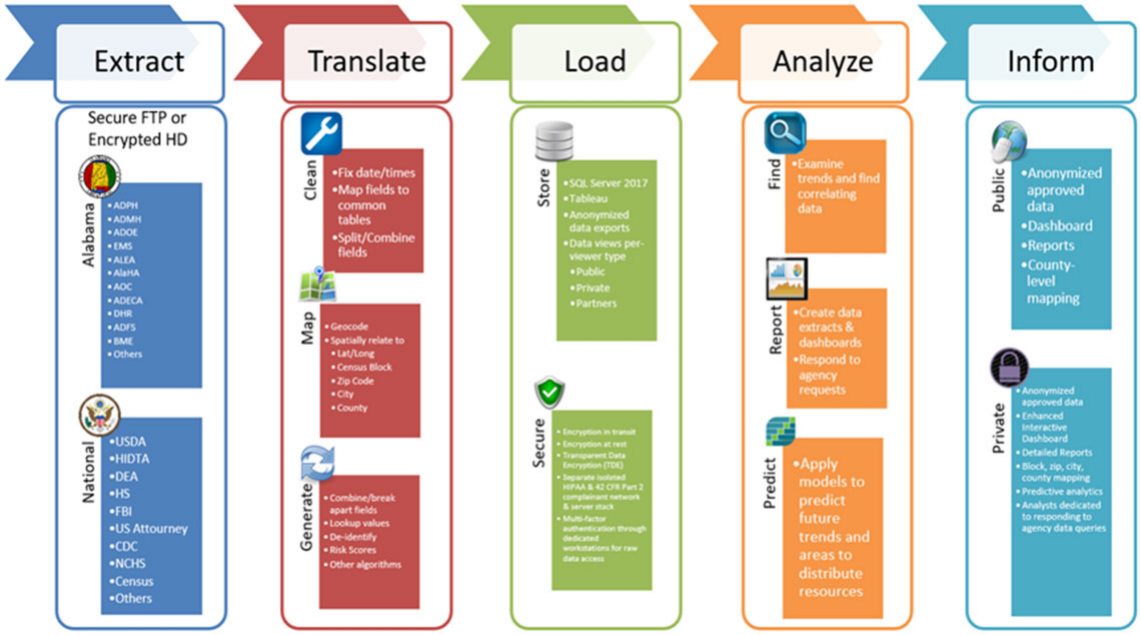
DrugUse-CDR Extract-Translate-Load (ETL) data flow.

Based upon the contractual data sharing agreements arranged with the IDA and each agency represented on DrugUse-CDR, the IDA uses custom ETL (ie, Extract, Translate, and Load) procedures to upload data into the repository. Figure 1, the DrugUse-CDR data flow, shows some of the many data sources that feed DrugUse-CDR and the steps performed at each level of the ETL process. Initially, the raw data for each participating agency is loaded to a server hosted by IDA and translated into a standardized format that supports analyses using data across participating agencies. Data for the CDR are imported in many formats, and if record-level data cannot be provided, the data are aggregated at the county level on ideally monthly or at worst case yearly levels. These 2 parameters, a location and a timeframe, enabled the CDR to perform geo-temporal correlations between disparate datasets. In other cases where identifiable record-level information was available, data are linked through identifiers like SSN, DL, or Name/DoB/Address between datasets with tiered matching procedures. To reconcile data inconsistencies, we found the ideal approach is to provide both data and denote the differences in reporting requirements and statutory responsibilities between agencies, as often data would be similar but not completely matching. This is the case with EMS overdose runs and hospital-denoted overdoses since not all EMS runs end up at a hospital, and not everyone showing up to a hospital for an overdose is brought there by an ambulance.

After the data are translated, the data then flows into the load step of the ETL process. During the load phase, the data are persisted to disk. In compliance with HIPAA/HITECH, data in DrugUse-CDR are stored in Microsoft SQL Server with Transparent Data Encryption (TDE) enabled. TDE allows for the writing of data to a SQL server to be automatically encrypted on disk so that if the disk were ever stolen, the data itself would not be readable. Encrypted data at rest are a requirement of multiple federal regulations is a key tenet of the OpioidCDR architecture. Also, during the load phase, data are stored according to an attribute access control structure that ensures only those with appropriate access rights can view the corresponding data. All raw data access is also protected by 2-factor authentication, multiple tiers of segmented (firewalled) access, and all servers, workstations, and data processing machines are isolated in a HIPAA-compliant physical and logical network segment that is separate from all other campus connectivity.

After the data processes through the ETL phases, it is then made available to an DrugUse-CDR analyst who has been trained on the respective statutes and MOUs that govern the data. The analysts effectively “air-gap” the data repository in that no external connectivity to the backend repository could ever be made. This is a best-practice concept regularly used by military/intelligence organizations for maximum data protection as physical access to machines that can query the data are required, thereby preventing external hackers from ever potentially breaching the repository. Analysts have to VPN into terminal servers to extract the data, and not until they are disconnected are they able to again connect to the internet (no split-tunneling allowed). The terminal servers they connect to have access to the data with analytics tools on the terminals, but these systems do not have internet access. Exports from the analytics tools are transferred to the analyst’ machines, where they are then loaded to Tableau.com or disseminated otherwise after they disconnect from the remote systems. From a governance perspective, the process in place is that no information from any participating agency is ever used or disseminated without review and approval from a designated agency representative. While this process impacts turnaround time, it helps to ensure that the data meets the requirements and usage expectations of the partner agencies.

**Figure 2.**
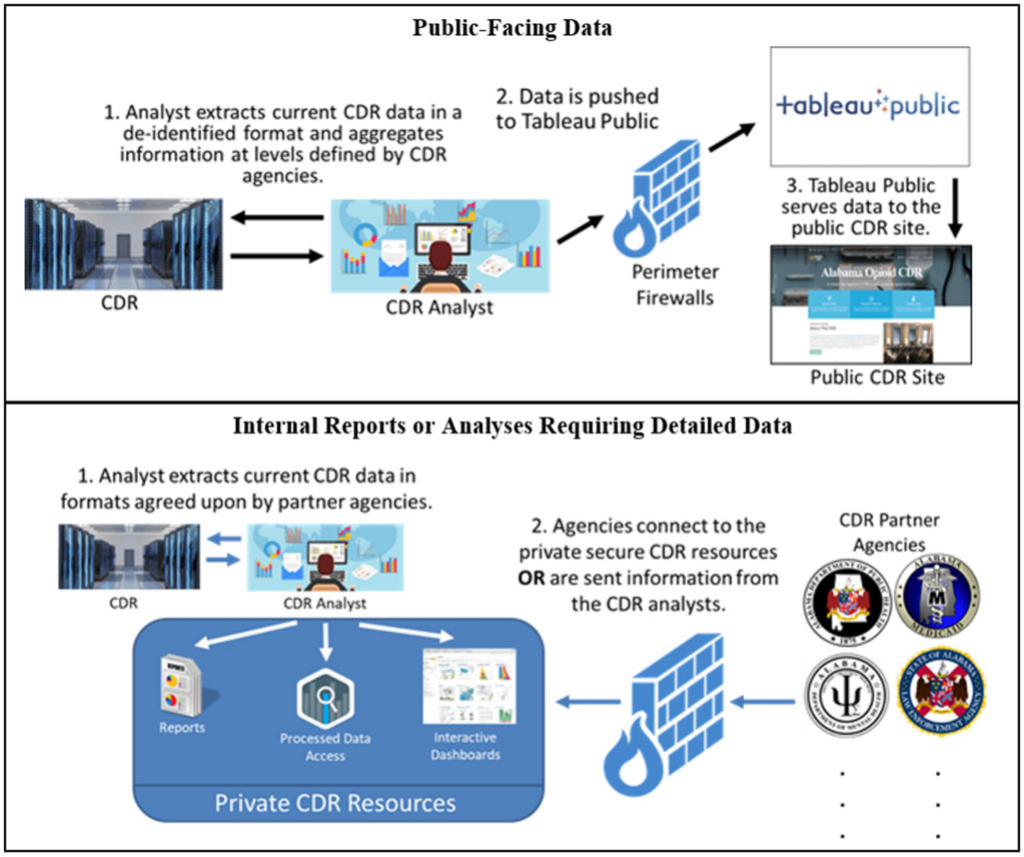
DrugUse-CDR data management flow.

The analysts extract information from the DrugUse-CDR into 2 distinct data formats, one that is public-facing and the other that is available with more detail back to the partner agencies (Figure 2). On the public side, de-identified data are extracted and aggregated, typically at the county level. For situations where ten or fewer records for a given region are available, these numbers are suppressed in keeping with CDC (Centers for Disease Control and Prevention) recommended guidelines.^10^ The data are then pushed by the analyst to Tableau Public © where researchers, policymakers, and the public can readily access the de-identified data.

**Figure 3.**
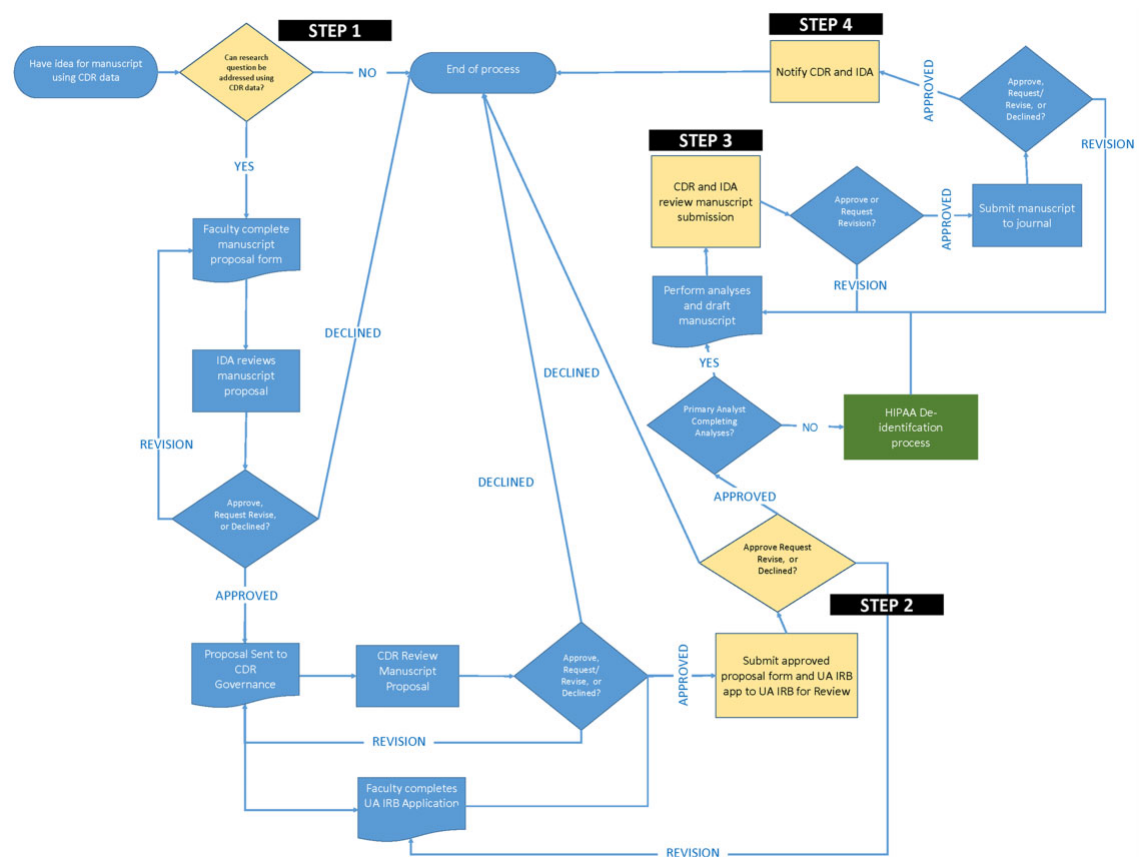
Research process.

Having a well-formalized process to vet and approve research questions is key to unearthing trends and gaining new insights for better policies (Figure 3). The established process flow allows for researchers to propose new ideas and for the affiliated stakeholders, data owners, and IRB to all vet the idea to ensure that the sensitive information within the repository is protected while still providing opportunities for expanded research. For research and internal reports, DrugUse-CDR analysts extract data from the repository and load the data into a private secured server. Partner agencies securely connect to the private repository using a VPN with 2-factor authentication to remote terminal servers with a secondary 2-factor authentication check. Through these terminals, analysts from the partner agencies can view and interact with optimized slices of their data. The segmented environment prevents unauthorized access and limits agencies to only their data that has either been cleaned, augmented with other public/purchased data, or data that has been explicitly permitted for their use by another agency. This prevents situations like law enforcement from accessing substance abuse information that would be in violation of 42 CFR Part 2 or other statutes. Holding to the principles of HIPAA-HITECH, data are de-identified when it does not significantly compromise the integrity of analyses, so stakeholders only have access to information supportive of tasks approved by all relevant stakeholders.

## LESSONS LEARNED FROM DEVELOPING DRUGUSE-CDR

1. Data sharing agreements between government agencies is an important, but lengthy process

When considering data agreements for large-scale health data sharing initiatives, it is critical to understand the underlying laws that govern substance abuse and mental health (42 CFR Part 2), medical data (HIPAA/HITECH), criminal justice data (CJIS), and other sensitive data sources. In addition to federal laws, existing state laws may further bind or prevent open sharing of information, as most states with prescription drug monitoring programs (PDMPs) have state statutes that restrict the usage of the affiliated information. Despite strong verbal support, data sponsoring stakeholders often have additional regulations and restrictions, making contract negotiations between lawyers very time-consuming. So, it was not shocking that the first 18 months of DrugUse-CDR focused solely on creating data-sharing agreements. Nevertheless, it is important to note that scholars hoping to have similar state-sponsored arrangements should expect similar efforts in the early phases of their project.

2. To gain trust between academic and government partners, relationships must be nurtured

Though legal negotiations appeared cumbersome on the surface, they allowed the IDA to build relationships and gain the trust of government stakeholders. Previous experiences suggest that it can take 2 or more years to build a relationship where a pipeline of research can be facilitated. Though this may appear very labor-intensive for an academic scholar, the investment of time nurturing relationships have usually resulted in robust collaborations in the long term. The current governance structure of the CDR is made up of public health partners like the Alabama Department of Mental Health, Public Health, and Medicaid, law enforcement and judicial partners such as the Alabama Law Enforcement Agency, Pardons and Paroles, and the Department of Correction, private insurance partners like BlueCross BlueShield of Alabama (∼94% Alabama private insurance market share), and community partners such as Federally Qualified Health Centers (FQHCs) and community drug prevention and intervention organizations. Academic researchers from multiple domains and expertise are key members of the advisory board as they are often at the leading edge of grant writing, research, and exploratory interventions. Transportation, education, labor, and other agencies and organizations are on the plan to integrate into the CDR in the future as most entities have data and concerns that either track or are impacted by persons with substance use disorders.

3. There were unplanned disruptions in activities, which facilitated the improvement of DrugUse-CDR’s operations

Like many research projects in the United States, the COVID-19 pandemic presented challenges in daily operations. For a few months, our government stakeholders, as well as university research operations, were almost halted as the nation was figuring out how to carry out routine activities in the early stages of the COVID-19 pandemic. Though the COVID-19 pandemic presented challenges, it allowed DrugUse-CDR to develop activities that improved operations. The secure file transfer protocol (sFTP) was strengthened with enhanced security to the point where high-volume physical disk transfers between agencies and IDA were not needed. Also, video conferencing platforms facilitated increased communication between stakeholders.

4. Early assessments of data suggest that the driving force of the opioid epidemic is bigger than issues isolated to opioids

When DrugUse-CDR began, it was solely focused on the misuse and abuse of opioids in Alabama. As stakeholders started to examine data in the repository, it became clear that DrugUse-CDR should be equipped to handle the analyses of the misuse and abuse of all drugs. Not only are opiates often misused with other illicit drugs, but the social determinants of the misuse and abuse of many drugs are also very similar. Given this understanding, the IDA and stakeholders decided to restructure the data architecture to accommodate the analyses of various drugs.

## CONCLUSION

As drug misuse and abuse persist nationwide, time-sensitive analyses are necessary to address this issue. Stakeholders within academia and government are highly skilled in mitigating the opioid epidemic if given adequate tools (ie, data) to help fix the problem. We hope that DrugUse-CDR can serve as a start to similar data-centric collaborations in the state of Alabama, where government agencies are able to leverage talents across stakeholders, thereby improving the efficiency and human capital within each agency’s daily operations.

## FUNDING

This work was supported by the Alabama Department of Mental Health under grant number A19-0167-001, which was funded through US DOJ Bureau of Justice Assistance (BJA) grant number 2018-AR-BX-K018. The manuscript was also directly through US DOJ Bureau of Justice Assistance (BJA) grant number 2018-AR-BX-K097.

## AUTHOR CONTRIBUTIONS

All of the authors contributed to the conception and design of the work, data acquisition, analysis, and interpretation. All authors also contributed to the drafting of the manuscript, content, and revisions.

## CONFLICT OF INTEREST STATEMENT

None declared.

## Data Availability

The public-facing CDR is available with many forms of data at https://druguse.alabama.gov. Identifiable data for research purposes must be vetted by the agency(s) that sourced the data, and requests for access can be made through petitions from any of the authors.
